# Whole-body MRI in children aged 6–18 years. Reliability of identifying and grading high signal intensity changes within bone marrow

**DOI:** 10.1007/s00247-022-05312-y

**Published:** 2022-04-21

**Authors:** Pia Zadig, Elisabeth von Brandis, Paola d’Angelo, Laura Tanturri de Horatio, Lil-Sofie Ording-Müller, Karen Rosendahl, Derk Avenarius

**Affiliations:** 1 Department of Radiology, University Hospital of North-Norway, Tromsø, Norway; 2 Department of Clinical Medicine, Uit, The Arctic University of Norway, Tromsø, Norway; 3 Division of Radiology and Nuclear Medicine, Oslo University Hospital, Oslo, Norway; 4 Faculty of Medicine, Institute of Clinical Medicine, University of Oslo, Oslo, Norway; 5 Department of Pediatric Radiology, Ospedale Pediatrico Bambino Gesù, Rome, Italy

**Keywords:** Adolescents, Bone marrow, Children, Magnetic resonance imaging, Validation study, Whole-body imaging

## Abstract

**Background:**

Whole-body magnetic resonance imaging (MRI) is increasingly being used in children, however, to date there are no studies addressing the reliability of the findings.

**Objective:**

To examine intra- and interobserver reliability of a scoring system for assessment of high signal areas within the bone marrow, as visualized on T2-weighted, fat-saturated images.

**Materials and methods:**

Ninety-six whole-body MRIs (1.5 T) in 78 healthy volunteers (mean age: 11.5 years) and 18 children with chronic nonbacterial osteomyelitis (mean age: 12.4 years) were included. Coronal water-only Dixon T2-weighted images were used to score the left lower extremity/pelvis for high signal intensity areas, intensity (0–2 scale), extension (0–4 scale) and shape and contour in a blinded fashion by two pairs of radiologists.

**Results:**

For the pelvis, grading of bone marrow signal showed moderate to good intra- and interobserver agreement with kappa values of 0.51–0.94 and 0.41–0.87, respectively. Corresponding figures for the femur were 0.61–0.68 within and 0.32–0.61 between observers, and for the tibia 0.60–0.72 and 0.51–0.73. Agreement for assessing extension was moderate to good both within and between observers for the pelvis (k = 0.52–0.85 and 0.35–0.80), for the femur (0.52–0.67 and 0.51–0.60) and for the tibia (k = 0.59–0.69 and 0.47–0.63) except for the femur metaphysis/diaphysis, with interobserver kappa values of 0.29–0.30. Scoring of shape was moderate to good within observers, but in general poorer between observers, with kappa values of 0.40–0.73 and 0.18–0.69, respectively. For contour, the corresponding figures were 0.35–0.62 and 0.09–0.54, respectively.

**Conclusion:**

MRI grading of intensity and extension of high signal intensity areas within the bone marrow of pelvis and lower limb performs well and thus can be used interchangeably by different observers, while assessment of shape and contour is reliable for the same observer but is less reliable between observers. This should be considered when performing clinical trials.

## Introduction

Whole-body magnetic resonance imaging (MRI) is currently used for several indications in children, particularly for the assessment of multifocality in inflammatory or malignant disease, and for the detection of preclinical or so-called “silent,” lesions. However, to date there is no consensus on a standardized imaging protocol or scoring system, and studies addressing the clinical validity of the MRI findings are lacking [1]. Moreover, normal growth and maturation of the skeleton, such as ossification and conversion of red hematopoietic marrow to yellow fatty marrow [2–4], affect the appearance of MR images. Previous studies have shown that up to half of healthy children ages 6–16 years have bone marrow edema-like changes in the hands and feet that might be mistaken for pathology [5–7]. This highlights the need for normal MRI references, and for precise tools to report on signal intensity variation in the bone marrow and its surrounding structures.

Several scoring systems for whole-body MRI in children have been suggested, none of which consider the whole spectrum of findings, from growth- and maturation-related findings to signal intensity changes caused by true disease [8, 9]. Ideally, an MRI score for structural change should reflect the total burden of disease damage only, thus excluding growth-related changes. To date, however, there is no unifying definition of true pathological bone marrow signal intensity on MRI [1]. The aims of the present study were to examine the intra- and interobserver reliability in the assessment of bone marrow signal intensity as seen on whole-body MRI in children and to suggest a novel scoring system based on the most robust features.

## Materials and methods

This study is part of a longitudinal, prospective multicenter study including 196 healthy volunteers and 40 patients with nonbacterial osteomyelitis, aiming to establish a new, MRI-based scoring system for whole-body MRI, to describe signal intensity variations in healthy children and to define differences between normal variation and true disease.

For this study, we included a subset of whole-body MRI examinations from 78 healthy volunteers and 18 patients diagnosed with chronic nonbacterial osteomyelitis between 6 and 18 years of age, examined at Oslo University Hospital between March 2018 and December 2019. The criteria for chronic nonbacterial osteomyelitis were mono- or multifocal inflammatory bone lesions (osteomyelitis, osteitis, osteosclerosis); duration of symptoms > 6 weeks and exclusion of infection and malignancy, according to Girschick et al. [10]. The healthy volunteers were recruited via e-mail, direct invitations and announcements on social media. The project was approved by the Regional Ethics Committee (REK; No 2016/1696) and written informed consent was obtained from all participants or their caregivers when appropriate.

The 96 included MR examinations reflected the whole spectrum of findings, to robustly test the variables within our MR-based scoring system. Areas hampered with motion artefact were excluded from further analysis.

### Magnetic resonance imaging

All whole-body MR examinations were acquired using a 1.5-Tesla (T) Magnetom Avanto fit system (Siemens Healthcare, Erlangen, Germany) and phased array surface coils. A total of five contiguous coronal stacks with T1-weighted, Dixon T2-weighted and diffusion-weighted images (b-values 0 and 800) from skull base to toes were performed with the participants supine and hands placed above the pelvis. No contrast agents or sedatives were given. The Dixon T2-weighted sequence was performed with an acquired voxel size of 0.9 × 0.9 × 3.5 mm, an echo time of 100 ms, a gap of 0.7 and 0% oversampling.

### Image review

For the present study, the water-only Dixon T2-weighted images were used. All images were read in a predefined room and window/level setting and scored independently by two pairs of radiologists, twice (at an interval of at least 4 weeks) by P.Z. and D.A. (with 5 and 20 years of experience, respectively) and once by E.v.B. and L.-S.O.-M. (both with 15 years of experience) without any additional information available. Before scoring, the observers calibrated their interpretation of the suggested protocol over two 2-day meetings and several video conferences, followed by consensus scoring of five whole-body MRI examinations from children not included in the present study. After an initial scoring of all right lower limbs and right hemi-pelvises, a final calibration process was performed, including minor adjustments of the scoring system and construction of an atlas with reference images to better define the different scores. Finally, the left lower extremities and the left hemi-pelvises were scored.

**Fig. 1:**
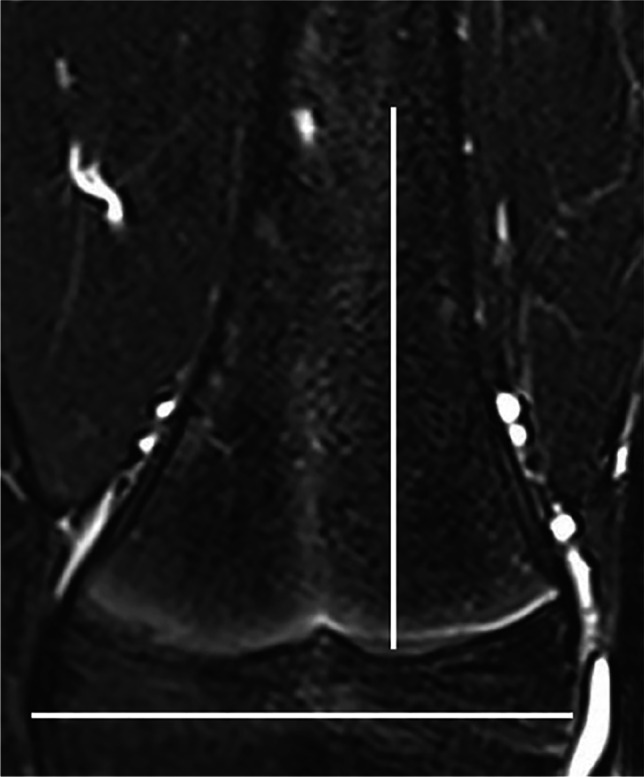
A coronal water-only Dixon T2-weighted magnetic resonance image in a 16-year-old girl with an area of increased signal intensity just within the borders of the metaphysis of the tibia, as calculated by the maximal width of the epiphysis

Eighteen anatomical regions were scored separately: central and lateral sacrum, ilium, ischiopubic and periacetabular region, trochanter major apophysis, proximal and distal epiphysis and metaphysis, and diaphysis of the femur and the tibia. The foot was divided into hindfoot (talus and calcaneus) and forefoot (the rest of the tarsal bones and the metatarsal bones). The size of the metaphysis was defined by a square over the growth plate of the affected bone, each side with a length equal to the maximum width of the epiphysis, except for the proximal femur metaphysis with the lower border defined by the lower border of trochanter minor [11] (Fig. 1).

**Fig. 2:**
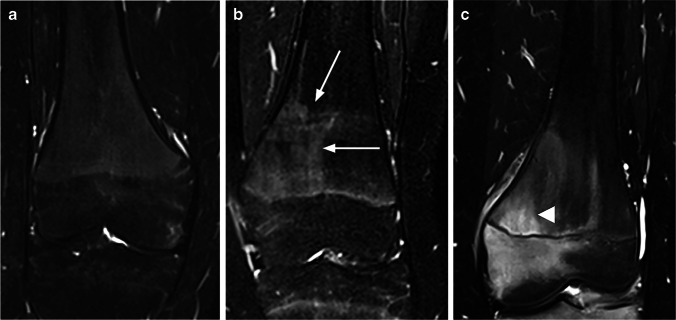
Coronal water-only Dixon T2-weighted magnetic resonance images in **(a)** a 16-year-old girl with only background signal intensity in the femoral metaphysis, **(b)** a 15-year-old girl with an area of high signal intensity grade 1 in the metaphysis of the femur (*arrows*) and **(c)** a 17-year-old boy with an area of high signal intensity grade 2 in the metaphysis of the femur (*arrowhead*)

**Fig. 3:**
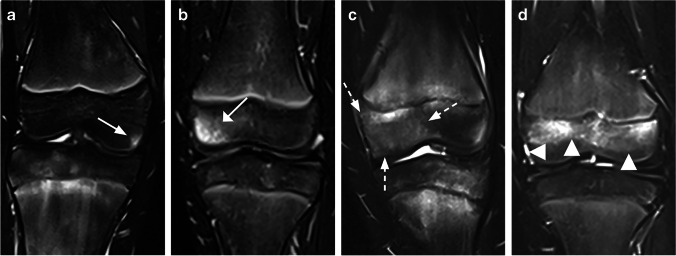
Coronal water-only Dixon T2-weighted magnetic resonance images in **(a)** a 14-year-old girl with an area of increased signal intensity in the epiphysis of the femur with an extension up to 5% (*arrow*), **(b)** a 7-year-old girl with an area of increased signal intensity in the epiphysis of the femur with an extension up to 1/3 (*arrow*), **(c)** a 14-year-old boy with an area of increased signal intensity in the epiphysis of the femur with an extension up to 2/3 (*dotted arrows*) and **(d)** a 7-year-old girl with an area of increased signal intensity in the epiphysis of the femur with an extension up to 3/3 (*arrowheads*)

**Fig. 4:**
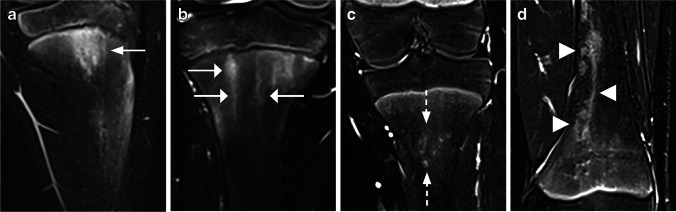
Coronal water-only Dixon T2-weighted magnetic resonance images in **(a)** an 11-year-old boy with a roundish area of increased signal intensity in the metaphysis of the tibia (*arrow*), **(b)** an 11-year-old boy with multiple areas of linear-shaped increased signal intensity in the metaphysis of the tibia (*arrows*), **(c)** a 12-year-old girl with an area of punctuated high signal intensity in the metaphysis of the tibia (*dotted arrows*) and **(d)** a 13-year-old girl with an area of increased signal intensity in the femur metaphysis with a mixed roundish and linear shape *(arrowheads*)

**Fig. 5:**
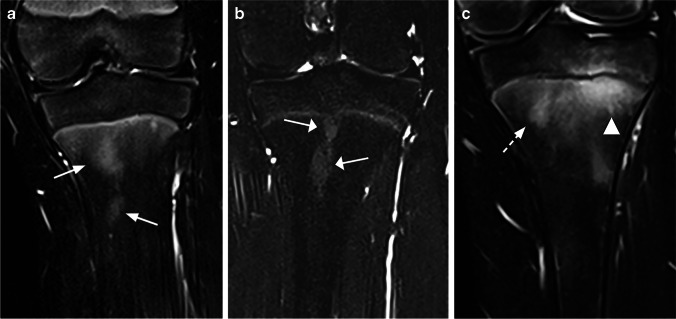
Coronal water-only Dixon T2-weighted magnetic resonance images in **(a)** a 10-year-old boy with an area of increased signal intensity in the metaphysis of the tibia with a diffuse contour (*arrows*), **(b)** a 16-year-old girl with an area of increased signal intensity in the metaphysis of the tibia with a sharp contour (*arrows*) and **(c)** a 14-year-old boy with an area of increased signal intensity in the metaphysis of the tibia with both a sharp and a diffuse contour (*dotted arrow* and *arrowhead*, respectively)

**Fig. 6:**
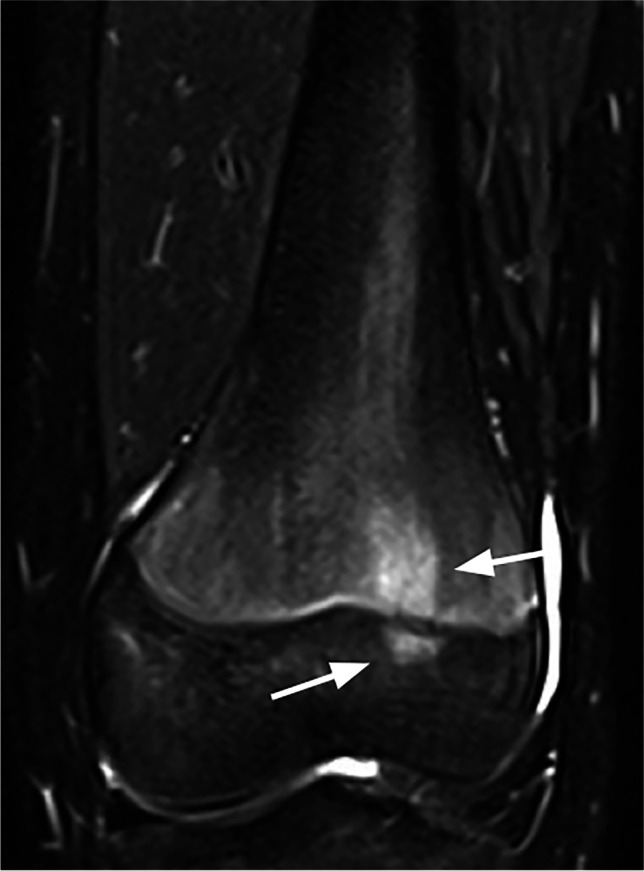
Coronal water-only Dixon T2-weighted magnetic resonance image in a 14-year-old girl with an area of high signal intensity with the same width on both sides of the physis scored as focal periphyseal edema (FOPE) (*arrows*)

Each of the anatomical regions were assessed for areas of high signal intensity in the bone marrow, which were scored for signal intensity (more than background signal) on a 0–2 scale (0 = absent/no high signal intensity area, 1 = mildly increased, 2 = moderately increased up to fluid-like signal) (Fig. 2), for extension on a 0–4 scale (0 = absent/no high signal area, 1 =  < 5% of the volume, and then increments of 1/3 of the subjectively perceived volume of bone segment) (Fig. 3), for shape (roundish, linear, combined roundish/linear or punctate) (Fig. 4), for the contour of the dominant part (sharp, diffuse or both) (Fig. 5) and for high signal intensity in the periosteum and/or adjacent soft tissue (no, yes). We also registered the presence of a high signal intensity line in the synovial part of the sacroiliac joint space (no, yes). The metaphyseal-equivalent high signal intensity in the sacrum was scored on a 1–4 scale, according to Chauvin et al. [12]. Focal periphyseal edema (FOPE), defined as an area of high signal intensity with the same width on both sides of the physis of long bones [13], was registered (no, yes) as a separate feature (Fig. 6). In addition, we scored the presence of speckled high signal intensity (multiple signal foci with a diameter less than 5 mm) in the foot on a 0–2 scale (0 = absent, 1 = sparse, 2 = extensive). Background signal in the diaphysis and metaphysis of femur and tibia, thin and/or punctate (less than 2 mm) high signal intensity areas in the epiphysis of femur and tibia, thin and vertical high signal intensity lines within the diaphysis (consistent with a vessel), and high signal intensity along the calcaneal apophysis were registered, but not further analyzed. Neither high signal intensity along the metaphyseal border of the epiphysis nor metaphyseal high signal intensity lines near the ends of long bones (due to bisphosphonate therapy), were scored.

### Statistical analysis

Differences in scoring were analyzed for each of the anatomical regions with the different features separately, using kappa statistics (Cohen's kappa for nominal variables and Cohen's weighted kappa for ordinal variables, with 95% confidence intervals [CI]). A kappa score of < 0.2 is considered poor, 0.21–0.40 fair, 0.41–0.60 moderate, 0.61–0.80 good and 0.81–1.00 very good [14]. The interobserver agreement was analyzed by comparing the scores of E.v.B. and L.-S.O.-M. with the second scores of P.Z. and D.A. Absolute interobserver agreement for each of the scores was calculated and given in percentages. Statistical analyses were performed using SPSS version 26.0 (IBM Corp. Released 2013; IBM SPSS Statistics for Windows, Armonk, NY).

## Results

**Fig. 7:**
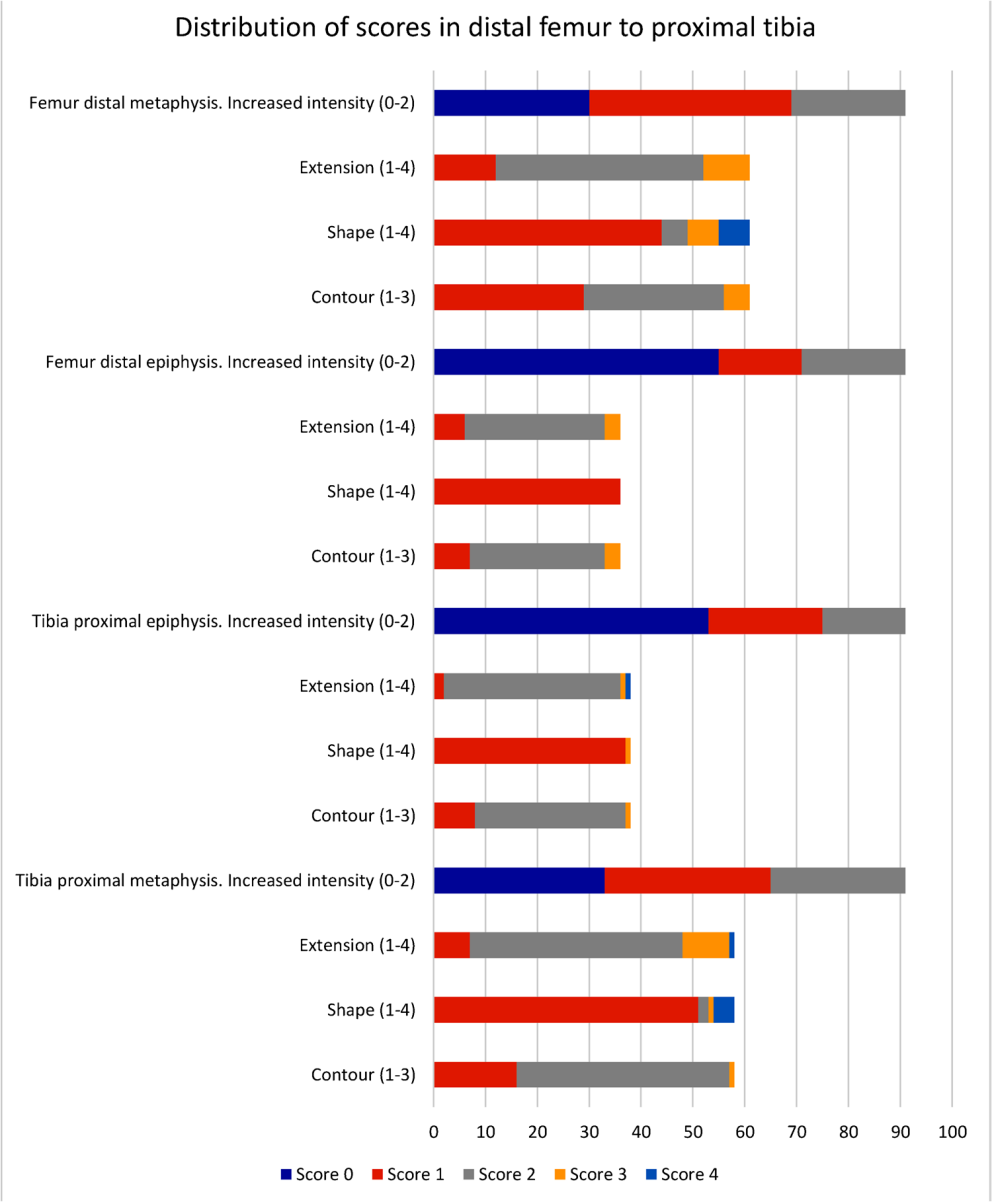
Distribution of scores for intensity, extension, shape and contour from the distal femoral to the proximal tibial metaphyses for the 96 whole-body magnetic resonance imaging examinations in children/adolescents ages 6–18 years

Ninety-six whole-body MR examinations from 78 healthy volunteers (38 females, mean age: 11.5 years [standard deviation (SD) 3.7]) and 18 individuals diagnosed with chronic nonbacterial osteomyelitis (5 females, mean age: 12.4 years [SD 2.9]) were included. None of the examinations, but 0–7 cases within each of the areas scored, was excluded from the analysis due to artefacts. Of the 96 children, 34 were under 10 years of age, 19 were 10–12 years, 24 were 13–15 years and 19 were 16–18 years of age. Preliminary analysis did not reveal significant differences in kappa values according to sex, thus, the results are presented for both sexes together. A total of 570 high signal intensity areas in the left hemi-pelvis/lower extremity were identified based on the first scoring session (P.Z. and D.A.): 110 in the pelvis, 225 in the femur, 172 in the tibia and 63 in the foot. The distribution of features/scores (intensity, extension, shape and contour) from the distal femoral metaphysis to the proximal tibial metaphysis is shown in Fig. 7, while the distribution of scores across all anatomical locations is shown in Online Supplementary Material 1.

### Pelvis

Grading of increased bone marrow signal intensity on a 0–2 scale in the lateral area of the sacrum, ilium and periacetabular area showed moderate to very good agreement both within and between observers, with kappa values of 0.51–0.94 and 0.41–0.87, respectively. For the ischiopubic region, the kappa values were 0.61 and 0.35 for intra- and interobserver agreement, respectively (Table 1). Likewise, the agreement for assessing the extension of areas with increased signal intensity performed well for the same observer, with kappa values of 0.52–0.85, while agreement between observers was slightly lower, with kappa values of 0.35–0.80 (Table 1). For shape and contour, the agreement for the same observer varied with kappa values of 0.33–0.78, while the agreement between observers was moderate for the lateral area of the sacrum, and fair for the ilium, the ischiopubic region and the periacetabular region (Table 1). The agreement for assessing Chauvin score was good within, and fair between, observers with kappa values of 0.58 and 0.32, respectively. A high linear signal in the sacroiliac joint space was noted in 64.2–70.5% of the examinations, and yielded kappa values of 0.33 within (absolute agreement 70.1%) and 0.31 between observers (absolute agreement 67.4%). The central sacral area showed too few signal intensity changes for statistical analysis to be performed.

**Table 1:** Pelvis. Cohen’s kappa values and proportions of absolute interobserver agreement for the assessment of intensity, extension, shape and contour on water-only Dixon T2-weighted images. There were 110 high signal intensity areas in 96 subjects who had whole-body magnetic resonance imaging

Region and imaging feature	Intra-observer kappa value (with 95% CI)	Interobserver kappa value (with 95% CI)	Interobserver proportion absolute agreement
Sacrum (lateral area)^*^			
- Intensity	0.94 (0.86–1.02)	0.87 (0.75–0.98)	94.7%
- Extension	0.85 (0.75–0.95)	0.80 (0.66–0.94)	93.7%
- Shape	0.78 (0.62–0.94)	0.64 (0.46–0.82)	88.4%
- Contour	0.74 (0.58–0.90)	0.60 (0.43–0.77)	89.5%
Ilium			
- Intensity	0.51 (0.28–0.73)	0.41 (0.18–0.63)	75.3%
- Extension	0.52 (0.30–0.75)	0.39 (0.16–0.62)	74.2%
- Shape	0.33 (0.14–0.52)	0.24 (0.06–0.42)	73.1%
- Contour	0.35 (0.15–0.55)	0.19 (0.03–0.35)	70.1%
Ischiopubic region			
- Intensity	0.61 (0.45–0.77)	0.35 (0.14–0.56)	65.3%
- Extension	0.55 (0.40–0.70)	0.35 (0.17–0.54)	60.0%
- Shape	0.55 (0.39–0.71)	0.27 (0.11–0.43)	64.2%
- Contour	0.51 (0.35–0.67)	0.16 (0.02–0.30)	57.9%
Periacetabular region			
- Intensity	0.65 (0.52–0.78)	0.47 (0.30–0.64)	67.0%
- Extension	0.53 (0.37–0.69)	0.44 (0.28–0.61)	59.6%
- Shape	0.53 (0.38–0.68)	0.36 (0.20–0.51)	67.0%
- Contour	0.49 (0.35–0.63)	0.22 (0.10–0.34)	55.3%
Chauvin	0.58 (0.44–0.73)	0.32 (0.19–0.45)	46.2%

^*^ The central sacral area showed too few signal intensity changes for statistical analysis to be performed. Linear weighted kappa used for intensity, extension and Chauvin grade. Simple Cohen’s kappa used for shape and contour. *CI* confidence interval

### Femur

Grading of increased bone marrow signal intensity on a 0–2 scale in the epiphysis, metaphysis and diaphysis showed good agreement for the same observer, with kappa values of 0.61–0.68, while agreement between observers was moderate to good except for the diaphysis and the distal metaphysis with a kappa value of 0.32 and 0.37, respectively (Table 2). The agreement for assessing the extension of increased signal intensity was moderate to good both within and between observers, except for the metaphysis with interobserver kappa values of 0.29 for the distal and 0.39 for the proximal metaphysis, and for the diaphysis with interobserver kappa value of 0.30 (Table 2). The agreement for assessing shape and contour was moderate to good for the same observer, and fair to moderate between observers.

**Table 2:** Femur. Cohen’s kappa values and proportions of absolute interobserver agreement for the assessment of intensity, extension, shape and contour on water-only Dixon T2-weighted images. There were 225 high signal intensity areas in 96 subjects who had whole-body magnetic resonance imaging

Region and imaging feature	Intra-observer kappa value (with 95% CI)	Interobserver kappa value (with 95% CI)	Interobserver proportion absolute agreement
Proximal epiphysis			
- Intensity	0.61 (0.32–0.89)	0.61 (0.33–0.88)	91.6%
- Extension	0.55 (0.25–0.84)	0.60 (0.35–0.85)	89.5%
- Shape	0.51 (0.22–0.80)	0.57 (0.31–0.83)	91.6%
- Contour	0.35 (0.11–0.59)	0.30 (0.12–0.48)	86.3%
Proximal metaphysis			
- Intensity	0.63 (0.48–0.78)	0.51 (0.34–0.67)	67.0%
- Extension	0.52 (0.37–0.68)	0.39 (0.23–0.55)	46.8%
- Shape	0.51 (0.35–0.67)	0.30 (0.16–0.44)	56.4%
- Contour	0.44 (0.29–0.59)	0.31 (0.16–0.46)	57.4%
Diaphysis			
- Intensity	0.68 (0.56–0.80)	0.32 (0.17–0.47)	53.9%
- Extension	0.52 (0.39–0.66)	0.30 (0.16–0.44)	44.9%
- Shape	0.40 (0.27–0.53)	0.18 (0.06–0.30)	41.6%
- Contour	0.33 (0.20–0.46)	0.27 (0.14–0.40)	49.4%
Distal metaphysis			
- Intensity	0.66 (0.53–0.79)	0.37 (0.22–0.51)	57.1%
- Extension	0.57 (0.45–0.69)	0.29 (0.16–0.43)	42.9%
- Shape	0.55 (0.42–0.68)	0.28 (0.15–0.41)	56.0%
- Contour	0.51 (0.39–0.64)	0.09 (0.00–0.18)	34.1%
Distal epiphysis			
- Intensity	0.66 (0.52–0.80)	0.52 (0.38–0.67)	67.0%
- Extension	0.67 (0.53–0.81)	0.56 (0.41–0.7)	67.0%
- Shape	0.65 (0.50–0.80)	0.51 (0.35–0.67)	74.7%
- Contour	0.52 (0.37–0.67)	0.42 (0.27–0.57)	68.1%
Trochanter major apophysis			
- Intensity	0.64 (0.44–0.83)	0.48 (0.24–0.72)	81.9%
- Extension	0.67 (0.48–0.87)	0.51 (0.29–72)	77.7%
- Shape	0.51 (0.32–0.70)	0.44 (0.22–0.66)	81.9%
- Contour	0.48 (0.29–0.59)	0.28 (0.11–0.45)	76.6%

Linear weighted kappa used for intensity and extension. Simple Cohen’s kappa used for shape and contour. *CI* confidence interval

### Tibia

The agreement both within and between observers for grading increased bone marrow signal intensity was good except for the proximal metaphysis, with a kappa value of 0.51 between observers (Table 3). Likewise, agreement for assessment of the extension on a 0–3 scale was good for the same observer, and moderate to good between observers. The agreement for scoring shape and contour was moderate for the same observer, with kappa values of 0.42–0.62 and 0.37–0.59, respectively. Corresponding figures for shape and contour between observers were 0.33–0.63 and 0.21–0.54, respectively.

**Table 3:** Tibia and foot. Cohen’s kappa values and proportions of absolute interobserver agreement for the assessment of intensity, extension, shape and contour on water-only Dixon T2-weighted images. There were 172 high signal intensity areas in the tibia and 63 high signal intensity areas in the foot in 96 subjects who had whole-body magnetic resonance imaging

Region and imaging feature	Intra-observer kappa value (with 95% CI)	Interobserver kappa value (with 95% CI)	Interobserver proportion absolute agreement
Proximal epiphysis			
- Intensity	0.72 (0.59–0.85)	0.70 (0.58–0.83)	80.2%
- Extension	0.68 (0.53–0.83)	0.63 (0.48–0.77)	75.8%
- Shape	0.62 (0.47–0.77)	0.63 (0.48–0.78)	81.3%
- Contour	0.54 (0.39–0.69)	0.54 (0.40–0.68)	75.8%
Proximal metaphysis			
- Intensity	0.60 (0.47–0.73)	0.51 (0.38–0.65)	62.2%
- Extension	0.59 (0.44–0.74)	0.48 (0.34–0.62)	52.2%
- Shape	0.42 (0.27–0.57)	0.33 (0.18–0.48)	60.0%
- Contour	0.37 (0.22–0.52)	0.21 (0.07–0.35)	50.0%
Diaphysis			
- Intensity	0.63 (0.49–0.76)	0.62 (0.48–0.76)	71.4%
- Extension	0.61 (0.48–0.74)	0.47 (0.32–0.61)	56.0%
- Shape	0.51 (0.37–0.65)	0.35 (0.22–0.48)	56.0%
- Contour	0.48 (0.34–0.62)	0.24 (0.10–0.38)	25.0%
Distal metaphysis			
- Intensity	0.72 (0.60–0.85)	0.73 (0.60–0.85)	78.8%
- Extension	0.69 (0.55–0.82)	0.63 (0.51–0.76)	69.4%
- Shape	0.61 (0.46–0.76)	0.57 (0.43–0.71)	72.9%
- Contour	0.59 (0.44–0.74)	0.48 (0.34–0.62)	67.1%
Distal epiphysis			
- Intensity	0.68 (0.50–0.86)	0.64 (0.46–0.82)	83.5%
- Extension	0.65 (0.47–0.82)	0.56 (0.38–0.75)	78.8%
- Shape	0.56 (0.38–0.74)	0.47 (0.29–0.65)	78.8%
- Contour	0.46 (0.29–0.63)	0.45 (0.39–0.73)	77.6%
Hindfoot			
- Intensity	0.80 (0.67–0.92)	0.64 (0.49–0.80)	79.3%
- Extension	0.68 (0.54–0.83)	0.60 (0.45–0.74)	73.2%
- Shape	0.73 (0.58–0.88)	0.69 (0.54–0.84)	85.4%
- Contour	0.62 (0.47–0.77)	0.52 (0.38–0.66)	73.2%
Forefoot			
- Intensity	0.34 (0.12–0.55)	0.58 (0.4–0.80)	79.7%
- Extension	0.42 (0.21–0.63)	0.68 (0.51–0.84)	81.0%
- Shape	0.34 (0.12–0.56)	0.57 (0.38–0.76)	82.3%
- Contour	0.33 (0.14–0.52)	0.28 (0.17–0.39)	67.1%
Speckled signal	0.75 (0.62–0.87)	0.65 (0.53–0.77)	73.2%

Linear weighted kappa used for intensity and extension. Simple Cohen’s kappa used for shape and contour. *CI* confidence interval

### Foot

The agreement for grading bone marrow intensity, extension, shape and contour for the hindfoot was good to very good both within and between observers, while the forefoot scored somewhat poorer (Table 3). Speckled signals in the foot were seen in the majority of cases, with high absolute agreement between observers, and a good kappa value both within and between observers.

### Focal periphyseal edema (FOPE)

A total of 46 FOPEs were seen, of which 27 were located around the proximal tibial physis, 13 in the distal femoral physis, 1 in the proximal femoral physis and 5 in the distal tibia. The agreement for diagnosing the FOPEs in the proximal tibial physis was very good both within and between observers, with a kappa value of 0.78 (CI = 0.64–0.93) for intra- and interobserver reliability.

### High signal intensity in the periosteum and/or adjacent soft tissue

High signal intensity in the periosteum and/or adjacent soft tissue was seen in 0–4 cases for each of the anatomical regions, hindering statistics.

## Discussion

We have identified a set of markers to describe the whole range of bone marrow changes as assessed on whole-body MRI in children and adolescents. The markers include the assessment and grading of increased bone marrow signal intensity on water-only Dixon T2-weighted images, as well as the extension, shape and contour of these signal intensity changes, all representing features of interest when assessing pathology.

In our scoring system, we included grading of bone marrow signal intensity, as we believe this might help differentiate normal variation from true pathology and evaluate the severity of pathology and treatment response in a clinical setting. Grading intensity on a 0–2 scale performed well overall for the same observer, particularly in the lateral sacrum and the periacetabular region. Agreement between observers was moderate to good, with an exception for the ischiopubic region, the femoral diaphysis and the distal femoral metaphysis, probably due to the inherit high content of red bone marrow and high background signal, underscoring the importance of thorough calibration before scoring. In a study of 45 children (41 with chronic nonbacterial osteomyelitis) with a median age of 11 years, Zhao et al. [9] reported a mean kappa value of 0.75 for scoring of signal intensity of the long bones on a 0–2 scale. However, their results are not directly comparable with ours as they applied a free-marginal version of kappa.

Subjective grading of signal intensity on fat-suppressed T2-weighted images, including the water-only Dixon T2-weighted technique used in the present study, is hampered by several difficulties. First, MR images depict relative signal intensities, thus rescaling is automatically performed by the scanner for each of the images. To adjust for this, we used a standardized window and level setting, we also standardized the room lights to the lowest possible background illumination. Second, an individual’s perception of signal intensity is relative and depends on the surrounding background signal within the image, as well as on the observer’s experience in reading MR images [15–18]. Particularly in children, with red marrow converting to yellow over time, starting in the peripheral bones and progressing centrally, the identification of high signal intensity areas beyond that of residual red marrow might be challenging. Within long bones, conversion begins in the epiphysis, followed by the mid diaphysis and then the metaphysis [19]. On MRI, areas of red marrow return a homogenously increased signal with feathery margins on T2 fat saturated images. To help exclude these areas from the high signal intensity areas identified and scored in the present study, we used an atlas including a series of MR images across age groups. Future methods for automated segmentation of bone marrow signal intensities using deep learning algorithms may help overcome some of the abovementioned obstacles.

In our study, we addressed one basic step toward a scoring system for whole-body MRI, namely reliability of identifying and grading bone marrow findings. In a recent review, Panwar and colleagues [20] describe their initial consensus-driven phase toward developing a whole-body MRI scoring system for screening juvenile idiopathic arthritis patients. Through Delphi surveys, experts within the field suggested that a coronal fluid sensitive sequence, either a short tau inversion recovery (STIR) or a fat-suppressed T2-weighted sequence with additional images for specific parts of the body, would suffice. Their whole-body MRI scoring system for juvenile idiopathic arthritis focused on the assessment of the inflammation in the joints and enthesis of the body. They suggest that a scoring system should include grading of high signal areas and grading of extension (as we have done). They conclude that further studies on reliability and responsiveness testing are warranted. In our study, assessment of signal extension on a 0–4 scale performed well for the pelvis and tibia. In the femur, it performed well for the same observer and slightly poorer between observers, particularly for the distal femoral metaphysis. This compares well with a study by Tanturri et al. [21] of 76 children ages 5–19 years with juvenile idiopathic arthritis, reporting on moderate to excellent agreement for the same observer and moderate to good agreement between observers, for grading the extension of bone marrow hyperintensity on a 0–3 scale [21].

Zhao et al. [9] found a low interobserver agreement for extension of marrow edema in the proximal and distal metaphysis of long bones. They suggested that this was due to difficulties in clearly identifying the border between the metaphysis and the diaphysis. In our study, we sought to overcome this difficulty by using the same definition of the metaphysis as Schneidmuller et al. [11], which is independent of the individual’s age and size. Our approach resulted in moderate to good agreement for the same observer, but poorer agreement between observers. We speculate that this might be due to the inhomogeneous background signal in this area, which makes it difficult to outline an area with increased signal. Of note is that the study by Zhao et al. [9] was designed to examine the agreement of MRI findings in a clinical setting, including 11 different observers with varying experience in MRI diagnostics. In contrast, we sought to examine the potential of an MRI scoring tool given optimal conditions, such as an extensive and equal experience between the two pairs of observers, meticulous calibration, and a large data set with a wide distribution of the different findings and scores.

Shape and contour have, to our knowledge, not been included in scoring systems for bone marrow changes on MRI. In general, moderate to good agreement was found for the same observer, whilst agreement between two observers was fair to poor despite meticulous calibration. Our atlas focused primarily on grading the signal intensities, and to a lesser extent on the shape and contour of the high signal intensity areas, which may in part explain the poorer agreement between observers. We incorporated these features with the intention to characterize focal signal intensity changes in more detail, particularly to differentiate between normal variation and true pathology. Thus, when using these features in clinical research, the images should be analyzed by the same reader (central reading). The clinical validity of these markers remains to be determined.

Others have examined intra- and interobserver reliability for assessing bone marrow change using different scores and statistical methods. For example, Hemke et al. [22, 23] looked at bone marrow signal intensity in the knee, using intraclass correlation, thus their results are not comparable to ours. Free marginal Kappa statistics was used in the paper by Zhao et al. [9] instead of the commonly used Cohen’s/Fleiss’ kappa [24]. Both approaches have their limitations; Cohen’s/Fleiss’ kappa being influenced by prevalence and bias, which can lead to the paradox of high agreement but low kappa value, while with the free marginal version, values vary as a function of the number of rating categories used [24]. To help identify a kappa paradox, we included the percentage absolute agreement between observers. Further, to elucidate the robustness of our kappa values, we presented the distribution of scores for each of the variables.

We acknowledge several limitations to our study. Firstly, there is the subjective nature of developing any scoring system with inherent biases in observers' past experiences and understanding of the factors required to score. To overcome this, we performed several calibration sessions, to better define the different scores and scales. Secondly, several of the parameters tested showed a severely skewed distribution across categories, thus hindering estimation of kappa values. However, by adding the proportions of absolute agreement between observers we were able to identify variables with a high absolute agreement despite a moderate kappa value. Our study does not address the clinical validity of the scoring system; however, this was not our intention, which was to test the observer reliability of various features used to identify and quantify bone marrow changes.

The strengths of this study include the high numbers of subjects, assessment of both intra- and interobserver reliability and the inclusion of both normal variation and inflammatory lesions as well as the thorough calibration and development of an atlas prior to scoring.

## Conclusion

We have shown that grading of intensity and extension of high signal intensity areas within the bone marrow performs well in nearly all locations, and thus can be used interchangeably by different observers, while assessment of shape and contour is reliable for the same observer but is less reliable between observers. This should be considered when performing clinical trials.

## Declarations

This project was approved by the Regional Ethics Committee (REK; no 2016/1696) and written informed consent was obtained from all the participants or their caregivers when appropriate
